# Isoxanthohumol and Its Derivatives: Antioxidant Activity and Effects on the Gut Microbiota

**DOI:** 10.3390/molecules31081311

**Published:** 2026-04-17

**Authors:** Renata Choińska, Justyna Nasiłowska, Adrian Wojtczak, Włodzimierz Lewandowski, Renata Świsłocka

**Affiliations:** 1 Department of Fermentation Technology, Prof. Waclaw Dabrowski Institute of Agriculture and Food Biotechnology-State Research Institute, Rakowiecka 36, 02-532 Warsaw, Poland; renata.choinska@ibprs.pl; 2 Department of Microbiology, Prof. Waclaw Dabrowski Institute of Agriculture and Food Biotechnology-State Research Institute, Rakowiecka 36, 02-532 Warsaw, Poland; justyna.nasilowska@ibprs.pl (J.N.); adrian.wojtczak@ibprs.pl (A.W.); 3 Department of Chemistry, Biology and Biotechnology, Bialystok University of Technology, Wiejska 45E, 15-351 Bialystok, Poland; w.lewandowski@pb.edu.pl

**Keywords:** isoxanthohumol, xanthohumol, metal complexes, effective antioxidants, structure–activity, gut microbiota

## Abstract

Isoxanthohumol (IX) is a prenylated flavonoid derived from hop cones (*Humulus lupulus*) that is gaining increasing recognition for its potential biological effects. Despite numerous studies on its precursor, xanthohumol, studies on IX remain limited. Of particular interest is its metabolism, particularly its biotransformation by gut microbiota to 8-prenylnaringenin (8-PN), a potent phytoestrogen, which indicates the complex nature of its biological activity and potential health implications. This review summarizes the current state of knowledge on IX and its derivatives, covering their microbial metabolism, their impact on the gut microbiome, and the metabolic consequences of this conversion. Furthermore, it examines the relationship between the molecular structure of IX and its derivatives and their biological activity, highlighting existing research gaps and the need for further research on the safety and therapeutic potential of these compounds

## 1. Introduction

Isoxanthohumol (IX) is a prenylated flavonoid predominantly occurring in hops (*Humulus lupulus*) as a derivative of xanthohumol (XN), and has also been detected in extracts of *Sophora flavescens* roots [1,2]. Its chemical structure is thought to influence its reported antioxidant, estrogenic, anticancer, and antimicrobial activities, which have attracted interest because of their possible therapeutic relevance. Understanding the biosynthesis and metabolism of IX is thus crucial for both pharmacological studies and potential therapeutic applications. IX is not synthesized de novo but is mainly formed by the isomerization of XN. This conversion is a spontaneous or enzyme-facilitated intramolecular cyclization under certain conditions (e.g., heat during brewing or acidic environments). In plants, isomerization of XN is supported by chalcone isomerase (CHI) [1,3,4]. IX is metabolized in humans through cytochrome P450 enzymes (CYP1A2, CYP2C9, CYP3A4) and gut microbial enzymes to more active estrogenic compounds such as 8-prenylnaringenin (8-PN) [5]. Possemiers et al. [6] showed that *Eubacterium limosum* and other gut bacteria can O-demethylate IX into 8-PN. This transformation increases the estrogenic potency of IX post-ingestion. Its subsequent conjugation via UDP-glucuronosyltransferases (UGTs) and sulfotransferases (SULTs) catalyze glucuronidation and sulfation, respectively, to increase solubility for renal or biliary excretion [7,8]. Understanding these enzymatic pathways is important for interpreting the biological effects of IX and for assessing its possible pharmacological relevance. The biological activity of IX is closely related to its chemical structure, particularly the prenyl group, hydroxylation pattern, and flavanone backbone. These structural elements are thought to contribute to the reported estrogenic, antioxidant, anti-inflammatory, and anticancer activities of IX, while metabolic conversion to 8-PN may enhance selected biological effects. The biological activity of IX is lower than that of XN (due to the lack of an α,β-unsaturated ketone), although measurable effects have been reported in selected experimental models [9]. In in vitro studies, IX has been reported to inhibit proliferation and induce apoptosis in cancer cells, potentially through mechanisms such as cell cycle arrest, caspase activation, and mitochondrial dysfunction [10]. The antiproliferative activity of IX has been determined against various human cancer cell lines, including breast cancer (MCF-7), ovarian cancer (A-2780), prostate cancer (DU145 and PC-3), and colon cancer (HT-29 and SW620) [9,11]. IX has also been reported to induce apoptosis in human umbilical vein endothelial cells (HUVEC) and human aortic smooth muscle cells (HASMCs), suggesting potential anti-angiogenic properties. Furthermore, IX demonstrates antiviral effect against herpes viruses (HSV1 and HSV2) and bovine viral diarrhea virus (BVDV) [12]. However, the barrier to realizing the full potential of IX is its poor solubility and limited bioavailability [13]. One approach to addressing this issue is the formation of IX complexes, as it readily forms complexes with cyclodextrins, proteins (HSA), metals, lipids, and polymeric nanoparticles, which not only improve its physicochemical properties but also modulate IX’s biological activity. The most advanced applications include cyclodextrin inclusion complexes (nutraceutical delivery) and nanoparticle encapsulation (pharmacological studies).

This article analyzes the biological activity of IX and its derivatives in the context of their potential therapeutic applications, based on current knowledge of the relationship between the molecular structure of plant phenolic compounds and their antioxidant and biological effects [14,15]. At the same time, the complexity of IX’s action, including its microbiota-dependent metabolism, and its implications for efficacy and safety, are assessed. By highlighting these aspects, this work aims to contribute to a more comprehensive understanding of IX as a biologically active compound and to identify key directions for future research on its safe and effective use.

## 2. Microbial Metabolism of IX

The microbial conversion of IX into the more potent phytoestrogen 8-PN is an important contributor to the biological activity and bioavailability associated with hop-derived prenylflavonoids. Although this transformation has been recognized for nearly two decades, the specific bacterial taxa responsible for this reaction remain only partially characterized. Current knowledge suggests that a limited number of gut commensals possess the enzymatic capacity for O-demethylation of IX, and that interindividual variability in gut microbiota composition significantly influences the extent of this conversion.

For completeness, alternative microbial routes for IX metabolism (e.g., epoxidation/hydroxylation of the prenyl group and ring closures) have been reported, indicating metabolic branching beyond 8-PN [16]. The main enzymes, sites of action, and metabolites involved in the metabolism and biotransformation of isoxanthohumol are summarized in Table 1.

Among the intestinal bacteria studied to date, *E. limosum*—a rod-shaped, non-spore-forming, Gram-positive and strictly anaerobic species—is the most consistently and robustly identified species capable of converting IX into 8-PN. *E. limosum* exhibits remarkable metabolic versatility: it is commonly found not only in the human colon but also in other anaerobic environments such as the rumen and sediments, and it belongs to the group of acetogenic bacteria. This butyrate-producing Firmicute carries the enzymatic machinery necessary for O-demethylation, a reaction essential for activating IX. Experimental evidence from both in vitro fecal incubations and mono- and co-culture systems demonstrates the efficiency of *E. limosum* in catalyzing this reaction. Notably, in a foundational study by Possemiers et al. [20], this bacterium achieved conversion efficiencies of over 90% when incubated with pure IX under controlled conditions. The mechanism of IX conversion in *E. limosum* appears to involve the Wood–Ljungdahl (WL) pathway, a versatile metabolic route common among acetogenic bacteria, which enables methyl group transfer and carbon fixation. Key enzymatic components that may be involved in this pathway include methyltransferases and corrinoid proteins, which collectively mediate the demethylation of IX. Optimization experiments confirmed that stationary-phase biomass, especially in mildly alkaline conditions (pH 7.8–8), facilitated rapid and near-complete conversion of IX to 8-PN, even when the bacterial cells were no longer actively dividing [21]. Furthermore, co-culture and supplementation studies demonstrate that the addition of *E. limosum* to fecal suspensions from individuals lacking IX-converting microbiota can restore the conversion capacity. Even 1% (*v*/*v*) supplementation led to significant 8-PN production within 24 h, indicating not only the high catalytic efficiency of *E. limosum* but also its potential utility in probiotic or bioaugmentation strategies [22]. In contrast to *E. limosum*, *Eubacterium ramulus* does not contribute to the activation of IX. Instead, it appears to play an antagonistic role by degrading 8-PN through hydrogenation and O-demethylation, thereby reducing its estrogenic potential. In vitro studies show that *E. ramulus* transforms 8-PN into less active metabolites such as O-desmethylxanthohumol (DMX) and O-desmethyl-α,β-dihydroxanthohumol (DDXN). These dihydrochalcones lack the structural features necessary for estrogen receptor binding, and their formation effectively lowers the bioactive pool of 8-PN [23]. Interestingly, co-culture experiments involving both *E. limosum* and *E. ramulus* reveal a sequential metabolic interaction: *E. limosum* first demethylates IX into 8-PN, which is subsequently reduced by *E. ramulus* into DDXN. As a result, although 8-PN is produced, its accumulation is limited due to downstream metabolism. This highlights the importance of not only identifying IX-activating strains but also accounting for the presence of bacteria capable of further degrading phytoestrogenic metabolites [23].

**Figure 1:**
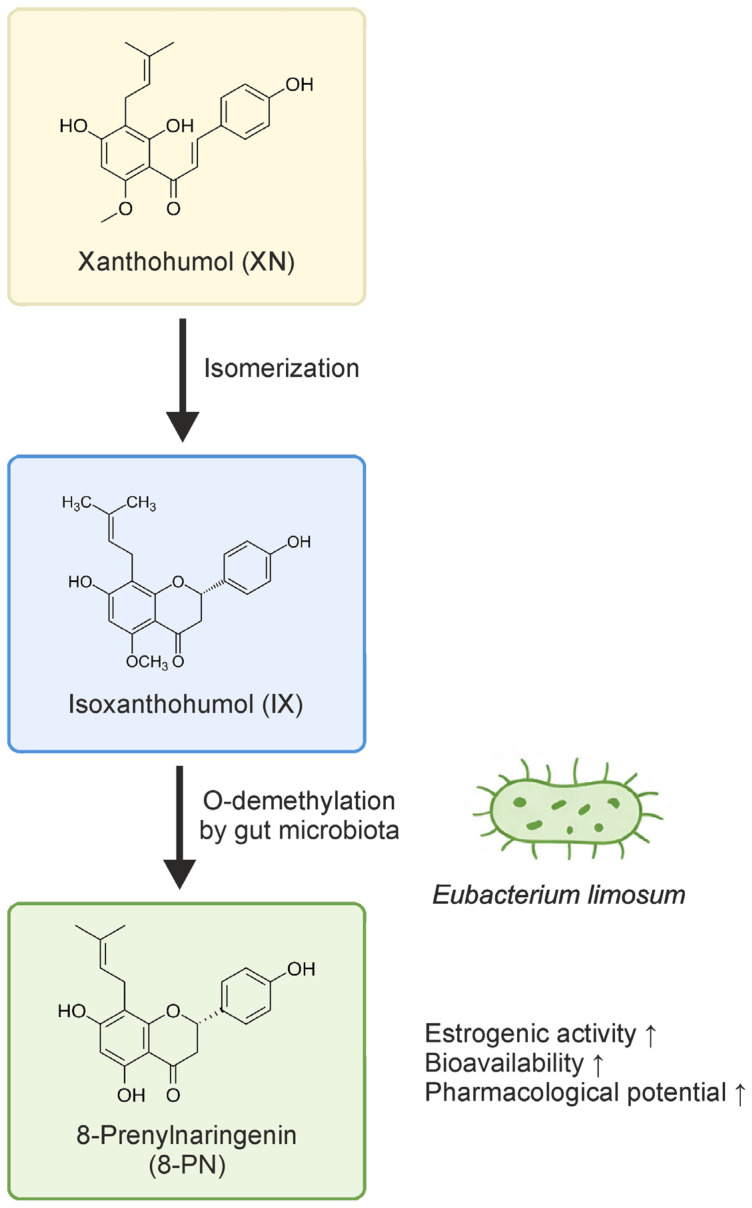
Conversion of Xanthohumol to 8-Prenylnaringenin via Isoxanthohumol.

While *E. limosum* remains the most well-characterized IX-converting bacterium, there is suggestive evidence that other taxa, particularly within the Firmicutes phylum, such as *Clostridium* spp. and Lachnospiraceae, may possess O-demethylating capacity. However, their roles are less clearly defined. Some fecal cultures demonstrate IX conversion despite the absence of detectable *E. limosum*, implying the involvement of alternative species or strain-level variation within broader taxonomic groups [21]. Interindividual variability is a critical factor in this context. Possemiers et al. [22] observed that only one-third of human fecal samples incubated with IX produced detectable levels of 8-PN, despite identical in vitro conditions. This suggests that the presence or absence of specific functional strains may be an important determinant of bioconversion potential, possibly more so than broad compositional features of the microbiota. Such findings are supported by in vivo studies in human microbiota-associated (HMA) rats, which also show significant variation in 8-PN excretion depending on the donor microbiota. The conversion of xanthohumol to 8-prenylnaringenin via isoxanthohumol is illustrated in Figure 1.

The identification of bacterial species involved in IX transformation relies heavily on advanced anaerobic culturing techniques, dynamic gut models (e.g., SHIME), and in vivo validation in gnotobiotic animal systems. Together, these methods have helped clarify not only which bacteria are responsible for this transformation but also the conditions under which they are active. For instance, suppression of IX conversion in the presence of certain hop-derived antibacterial compounds, or under suboptimal pH or redox conditions, underscores the complexity of translating these microbial reactions into predictable host-level effects [21]. Taken together, current evidence indicates that while *E. limosum* is the primary microbial agent capable of activating IX into 8-PN. Its activity is contingent upon multiple ecological and physiological factors. The presence of other bacteria, such as *E. ramulus,* that counteract this conversion further complicates the net outcome in a given host. Future research will benefit from more refined identification of strain-level differences and exploration of microbial consortia that either promote or inhibit 8-PN production.

## 3. Structure–Activity Relationship of IX and Its Derivatives

Structural differences between prenylated flavonoids influence their biological activity, molecular interactions, stability, and metabolism. The presence of a prenyl group at C-8 increases lipophilicity, which improves membrane permeability and cellular uptake while also enhancing binding to the estrogen receptor (ER) [24]. This allows prenylated compounds such as IX and especially 8-PN to exhibit significantly more potent estrogenic and anticancer effects compared to nonprenylated analogues such as naringenin [1,25]. The distinction between the flavanone (IX) and chalcone (XN) skeletons also leads to important functional differences: flavanones, possessing a saturated C2–C3 bond, are more stable but less electrophilic, whereas chalcones possess a reactive α,β-unsaturated carbonyl group. This α,β-unsaturated carbonyl group confers chalcones with electrophilic Michael acceptor properties, enabling covalent modification of nucleophilic residues (e.g., cysteines in Keap1) and subsequent activation of cytoprotective pathways such as Nrf2/Keap1. The ability of chalcones to scavenge free radicals, in turn, stems from the presence of hydroxyl groups, which act via redox mechanisms. Therefore, these two properties are mechanistically distinct but may act synergistically, simultaneously modulating cell defense pathways and directly neutralizing reactive molecules [9,26,27]. Substituents further modulate activity: the O-methyl group at C-7 in IX reduces ER binding compared to 8-PN, in which free hydroxyl groups are crucial for potent ER activation. Furthermore, in IX, O-methylation of the hydroxyl group at C-7 reduces its ability to donate hydrogen, whereas XN retains more accessible hydroxyl groups that support hydrogen transfer and radical stabilization. The presence of OH groups at C-5, C-7, and C-4′ plays a key role in antioxidant activity through hydrogen donation and radical scavenging, and activity varies depending on their position and accessibility. A study by Tošović et al. [27] on the radical scavenging profiles of IX and XN showed that XN exhibited a broader antioxidant potential, effectively neutralizing radicals such as hydroperoxyl and methoxyl by transferring hydrogen atoms and forming radical adducts. In contrast, IX exhibited a more limited activity, demonstrating efficacy mainly against the methoxyl radical. Metabolism can further alter the activity of prenylated flavonoids: demethylation of IX to 8-PN significantly enhances estrogenic potential, rendering the metabolite significantly more biologically active than its precursor. Finally, glycosylation increases solubility but reduces absorption, effectively delaying activity until bacterial hydrolysis in the gut, where the glycosides can act as prodrugs. Moreover, recent literature data, including authors’ works, indicate that antioxidant efficacy is determined not only by the number and position of hydroxyl groups in aromatic rings but also by the ease of proton and electron dissociation [28,29,30]. Other effects are also important, such as: (a) the degree of delocalization of the electronic charge distribution in antioxidants, (b) resonance stabilization of the molecule, (c) reduction in its energy, (d) elongation of the conjugated double bond system, and (e) complexation of antioxidants with metals with high ionic potential, such as Al(III), lanthanides(III), Fe(III), or Cr(III). Considering the full range of factors influencing the final result provides comprehensive information on the antioxidant efficacy of a given compound. The above studies and findings are extremely important because they can contribute to the effective search and design of new, effective antioxidants. In the context of IX, structural features such as the prenyl group and hydroxyl groups are crucial for both radical scavenging and metal chelating properties. Modifications of these groups in derivatives can regulate the balance between direct antioxidant activity and metal-binding capacity, thus influencing their pharmacological profile and potential therapeutic applications.

### Complexes of IX and Derivatives

Prenylated flavonoids, due to their specific structure, can form complexes that improve their physicochemical and biological properties. Cyclodextrin inclusion complexes, typically formed with β-cyclodextrin or hydroxypropyl-β-cyclodextrin, encapsulate hydrophobic molecules, significantly enhancing water solubility, photostability, and bioavailability. These systems are the most extensively studied and find wide application in nutraceuticals and food formulations [31,32,33]. IX noncovalently binds to human serum albumin or casein, forming protein complexes that stabilize the flavonoid in plasma, prolong systemic circulation, and alter tissue distribution, thereby influencing pharmacokinetics and promoting systemic drug delivery [34]. Complexes with lipid carriers, including liposomes, micelles, and nanoemulsions, protect the compounds from metabolic degradation, increase solubility, and improve intestinal absorption, making them suitable for both oral and parenteral administration. Studies suggest that IX and its derivatives can form complexes with transition metals, which influences their redox and enzymatic activity. However, further research is needed to better understand the mechanisms of these interactions and their potential therapeutic applications [31]. Furthermore, complexation of IX with polymeric and nanoparticle complexes based on materials such as PLGA, chitosan, or PEG has been investigated [33,35]. These complexes offer improved stability, controlled release, and potential tissue targeting, making them promising for preclinical anticancer and chemopreventive applications. Enzyme complexes and microbiota are also important because gut microbial O-demethylases convert IX to 8-PN, a significantly more potent phytoestrogen, highlighting the important role of biotransformation in vivo.

## 4. Impact of IX on Gut Microbiota

Recent research indicates that IX not only undergoes transformation by gut microbes but also actively influences the composition of the gut microbiota itself [6,7,8,9,10,11,12,13,14,15,16,17,18,19,20,21,22,23]. While most studies on interactions between flavonoids and microbiota have focused on mutual metabolic processes, an increasing body of evidence highlights IX’s more proactive, modulatory impact on the gut microbial environment. This interaction holds particular functional importance in relation to inflammation and metabolic imbalances.

A landmark study by Yamashita et al. [36] demonstrated that when mice fed a high-fat diet (HFD) were supplemented with IX, notable changes in gut microbiota composition occurred, alongside improvements in metabolic health indicators. Specifically, IX supplementation led to a substantial increase in the levels of *Akkermansia muciniphila*—a bacterium that breaks down mucin and is linked to metabolic balance and enhanced gut barrier integrity. At the same time, there was a reduction in *Clostridium* cluster XI. These shifts in microbial populations were accompanied by lower concentrations of lipopolysaccharide (LPS) in the blood, as well as decreased levels of pro-inflammatory cytokines like TNF-α and IL-1β. This suggests that IX may help reduce chronic, low-grade systemic inflammation. Notably, these microbial alterations occurred without major changes in body weight, suggesting that IX may affect the gut microbiota independently of overt weight loss [36]. Based on these results, Watanabe et al. [37] carried out detailed mechanistic experiments to validate the microbiota-dependent nature of IX’s effects. In this investigation, mice treated with IX consistently exhibited increased levels of *A. muciniphila*. However, this effect was absent in antibiotic-treated and germ-free (GF) mice, providing strong evidence that the benefits of IX depend on the presence of native gut microbes. Furthermore, GF mice that received fecal transplants from IX-treated donors experienced similar benefits—including reduced fat accumulation and improved glucose handling—supporting the view that IX-induced changes in the microbiota may contribute to host metabolic improvement.

Concerning selectivity, IX appears to specifically encourage the growth of *A. muciniphila* while showing limited activity against selected bacterial taxa. It also reduced the relative presence of potentially harmful genera such as *Clostridium* and *Bacteroides*, implying a possible targeted antimicrobial action or competitive exclusion mechanism. Additionally, IX was found to upregulate the expression of mucin (*Muc2*) and tight junction proteins like claudin-1, pointing to a microbiota-mediated strengthening of the intestinal barrier [37]. The consistent rise in *A. muciniphila* across both studies is especially significant, given the bacterium’s recognized role in supporting gut health. Though it degrades mucin in the intestinal lining, this activity actually stimulates mucin production, helping to preserve epithelial integrity. Furthermore, it influences immune functions and enhances metabolic health in both animal models and humans [38]. IX’s ability to increase *A. muciniphila* in preclinical models suggests that it may have potential as a dietary modulator of the gut microbiota. Collectively, these studies make a strong case that IX directly affects gut microbiota beyond simply being metabolized by it. The recurring enrichment of *A. muciniphila*, along with the decline in inflammation-related microbes like *Clostridium* spp., suggests that IX could serve as a targeted microbiota-modifying compound with benefits for both metabolic and immune health. These findings emphasize the need to account for individual microbiota profiles and responsiveness when considering the physiological effects of IX and in designing future therapies based on prenylflavonoids.

## 5. Physiological and Pharmacological Implications

### 5.1. Estrogenic Activity of 8-PN

Among naturally occurring phytoestrogens, 8-PN stands out as one of the most potent in terms of its binding affinity to human ER and biological activity. Derived from the prenylation of naringenin, 8-PN is produced endogenously in humans via gut microbial O-demethylation of IX. This compound has garnered attention not only for its remarkable receptor affinity but also for its selective action across tissues, raising both therapeutic hopes and safety concerns in the context of hormone-sensitive conditions.

The foundational study by Milligan et al. [25] provided one of the first systematic evaluations of 8-PN’s endocrine potential. In a series of in vitro assays using recombinant human ER, 8-PN displayed a relative binding affinity (RBA) of approximately 0.1 to ERα and ERβ, when benchmarked against 17β-estradiol. This value far exceeded the RBA of other hop-derived flavonoids, including IX, which showed negligible receptor interaction. More critically, 8-PN was confirmed to act as a full agonist, activating estrogen-responsive promoters in both yeast assays and human Ishikawa endometrial cells. These results confirmed the strong estrogenic activity of 8-PN in cellular assay systems and supported its classification as a potent phytoestrogen. Yet receptor binding is only one aspect of estrogenicity. The true relevance of 8-PN lies in its in vivo behavior, which has been characterized by a complex balance between efficacy and selectivity. Using ovariectomized rat models, Humpel et al. [39] explored how 8-PN functions under conditions of systemic estrogen deficiency. Their findings revealed that subcutaneous administration of 8-PN prevented the loss of bone mineral density (BMD) typically induced by ovariectomy, thus confirming its physiological activity. Interestingly, these bone-preserving effects occurred without the proportional stimulation of uterine tissues, a hallmark side effect of estradiol. At doses where 8-PN restored BMD, uterine weights were increased only marginally—by approximately 10% of the estradiol reference—highlighting a favorable selectivity profile. This tissue-specific estrogenicity was further demonstrated in ERE-luciferase transgenic mouse models, where 8-PN preferentially activated reporter genes in the bone and prostate, while sparing the endometrium. Together, these results support the notion that 8-PN behaves as a selective estrogen receptor modulator (SERM)-like compound, a class of molecules known for their agonist activity in some tissues and antagonist or neutral behavior in others.

Translating these preclinical insights to the clinical setting, Erkkola et al. [40] conducted a randomized, double-blind, placebo-controlled trial in peri- and postmenopausal women. Participants received standardized hop extracts containing 8-PN at doses of 100 μg or 250 μg daily for 12 weeks. Menopausal symptoms were tracked using the Kupperman Index, a validated tool for assessing vasomotor and psychosomatic complaints. Results indicated a statistically significant reduction in menopausal symptoms, particularly in hot flashes and night sweats, compared to placebo. While no clear dose–response gradient emerged, the higher dose group showed a faster onset of symptom relief, aligning with the pharmacodynamic expectations derived from animal studies. Importantly, no adverse estrogenic effects—such as endometrial thickening or abnormal uterine bleeding—were observed during the study period, suggesting a potentially safe profile in short-term administration. This outcome echoed the selective effects noted by authors, further strengthening the translational validity of 8-PN’s profile.

However, estrogenic activity is not inherently beneficial in all physiological contexts. In a detailed comparative study, Overk et al. [41] evaluated the estrogenicity of 8-PN and its precursor IX in an in vivo study comparing the estrogenicity of IX, 8-PN, and hop extracts in ovariectomized rats. While 8-PN induced classic estrogenic responses—including increased uterine wet weight, vaginal cornification, and elevated progesterone receptor expression—IX remained largely inactive unless biotransformed. This distinction reinforces the central role of gut microbiota in modulating systemic estrogenic exposure. Moreover, the effective doses of 8-PN required to elicit uterine responses were significantly higher than those typically encountered in dietary contexts, indicating that substantial systemic exposure is needed before risks manifest clinically. These findings indicate that the in vivo estrogenic response to 8-PN is dose-dependent and that IX itself shows much weaker activity unless converted metabolically.

Interestingly, the estrogenic behavior of 8-PN is not strictly agonistic. In a study exploring interactions with other endocrine-disrupting compounds, Aichinger et al. [42] demonstrated that 8-PN antagonized the effects of the *Fusarium* mycotoxins zearalenone (ZEN) and α-zearalenol in human endometrial carcinoma cells. These toxins, which often contaminate cereal grains and possess potent estrogenic activity, were shown to stimulate estrogen-responsive gene expression. However, co-incubation with 8-PN attenuated this activation, suggesting a competitive or modulatory mechanism. This highlights the chemical context-dependence of 8-PN’s activity, where it may act protectively against stronger xenoestrogens through receptor occupancy or indirect signaling interference.

Nonetheless, the safety profile of 8-PN is not without concern. A review by Minecka et al. [43] raised critical questions regarding the long-term use of hop-based preparations, particularly in populations with estrogen-sensitive disorders such as breast cancer, endometriosis, or uterine fibroids. The concern stems from the possibility of uncontrolled or prolonged estrogenic stimulation, especially when combined with efficient gut microbial conversion of IX to 8-PN. While the clinical data suggest minimal uterotrophic effects in short-term studies, longitudinal safety data remain limited, and interindividual variability in microbial metabolism may result in unpredictable systemic exposure to active compounds.

The final layer in understanding 8-PN’s pharmacological impact is its pharmacokinetics, particularly absorption, metabolism, and bioavailability. Using Caco-2 intestinal monolayer models, Nikolic et al. [44] demonstrated that 8-PN possesses good intestinal permeability but undergoes extensive phase I and phase II metabolism, primarily glucuronidation and sulfation. Enzymes such as UGT1A1, UGT1A6, and UGT1A9 catalyze these conjugations, significantly reducing the concentration of free 8-PN in circulation. However, this does not eliminate its activity. Tissues expressing high levels of β-glucuronidases (e.g., liver, reproductive organs, or tumor microenvironments) may locally deconjugate 8-PN, thereby reactivating its estrogenic potential. This compartmentalized pharmacology could explain why systemic plasma levels may underestimate the true biological activity of the compound, especially in tissues with active deconjugation or specific receptor profiles.

In summary, 8-PN emerges as a phytoestrogen of exceptional potency, with a distinct and multifaceted pharmacological profile. It combines high receptor affinity, tissue-selective activity, and context-dependent modulation, making it a compelling candidate for the treatment of menopausal symptoms and possibly osteoporosis. However, these same features also necessitate careful consideration in safety evaluations, particularly for individuals with high microbiota-driven conversion rates or predisposing conditions to hormone-sensitive pathologies. The future of 8-PN in therapeutic applications hinges not only on its proven benefits but also on our ability to predict and modulate its systemic exposure in a personalized, microbiota-aware framework.

### 5.2. Potential Health Benefits and Risks

As scientific interest in food-derived polyphenols and their interactions with the gut microbiota continues to expand, IX presents a particularly complex case. On the one hand, it exhibits potential for improving metabolic health, while on the other, its estrogenic metabolite raises legitimate safety concerns. The following section outlines current knowledge of the health-related benefits and risks associated with IX, with emphasis on its metabolic effects, endocrine implications, and the gaps that must be addressed before its widespread use can be considered safe and evidence-based.

Preclinical studies suggest that IX may support metabolic homeostasis, particularly in the context of diet-induced obesity and insulin resistance. In murine models fed a high-fat diet (HFD), IX supplementation resulted in improved glucose tolerance, lower fasting glucose levels, and enhanced insulin sensitivity [37,45]. These metabolic improvements were consistently observed across multiple studies and appear to be dose-dependent. For example, Yamashita et al. [36] demonstrated that even low dietary doses of IX (0.01–0.1% in feed) significantly reduced insulin resistance markers in HFD-fed mice. This was accompanied by favorable modulation of inflammatory mediators, including decreased expression of TNF-α and IL-1β in adipose tissue, as well as lower circulating levels of lipopolysaccharide (LPS), a pro-inflammatory endotoxin associated with metabolic dysregulation. Similarly, the abovementioned Watanabe et al. [37] provided mechanistic insights suggesting that IX directly interferes with intestinal lipid absorption. In their study, IX administration reduced the activity of pancreatic lipase and downregulated the expression of fatty acid transporters such as CD36 in the small intestine. This contributed to decreased triglyceride uptake and improved plasma lipid profiles. Of particular interest was the observation that IX-induced metabolic benefits were nullified when mice were treated with broad-spectrum antibiotics, strongly implicating microbiota-dependent mechanisms in the observed outcomes. Nevertheless, the metabolic improvements in the IX-treated group remained robust in antibiotic-free settings, emphasizing the compound’s intrinsic potential as a metabolic modulator. Notably, these effects extend beyond glucose metabolism. Miranda et al. [46] evaluated the effects of two hydrogenated derivatives of IX, i.e., α,β-dihydro-isoxanthohumol (DXN) and tetrahydro-isoxanthohumol (TXN)—which cannot be converted into 8-PN and thus lack estrogenic potential. These derivatives not only mirrored IX’s ability to improve glucose handling in HFD-fed mice, but also improved spatial memory and cognitive performance in behavioral assays. Moreover, DXN and TXN increased mitochondrial uncoupling in skeletal muscle cells, suggesting a mechanism of enhanced energy expenditure. The metabolic benefits observed in these models suggest that IX and structurally related compounds may be of interest for further investigation in metabolic disorders.

Despite these promising findings, the safety profile of IX is complicated by its bioconversion into 8-prenylnaringenin, a metabolite with potent estrogenic activity. Abovementioned work performed by Overk et al. [41] showed that while IX itself showed no significant estrogenic effects in terms of uterine weight gain or vaginal cornification in rats, administration of purified 8-PN at physiologically relevant doses elicited clear estrogenic responses. Uterine hypertrophy, upregulation of progesterone receptor expression, and changes in mammary gland histology were observed, closely mirroring the effects seen with estradiol. These findings underscore that even though IX is not directly estrogenic, its microbial metabolite can exert potent endocrine activity once formed in vivo. Further concerns arise from the study by Izzo et al. [47], which examined the effects of IX and 8-PN on steroidogenesis in primary cultures of rat Leydig cells. Both compounds significantly suppressed androgen production in response to human chorionic gonadotropin (hCG) across different developmental stages of Leydig cells. This suppression was associated with reduced intracellular cAMP levels and altered expression of key steroidogenic regulators such as the steroidogenic acute regulatory (StAR) protein. Importantly, these effects were not observed when steroidogenesis was stimulated with exogenous cAMP, suggesting a specific interference with receptor-level signaling. These results raise concerns about the potential for IX and 8-PN to disrupt hormonal regulation in the male reproductive system, particularly in adolescent or developmentally sensitive contexts.

The estrogenic potential of IX, through its conversion to 8-PN, introduces inter-individual variability that complicates risk assessment. Not all individuals possess the gut microbiota required to efficiently carry out this conversion, leading to substantial differences in circulating 8-PN levels following IX ingestion. In some cases, this could limit estrogenic exposure and reduce associated risks; in others, particularly among efficient converters, 8-PN accumulation could reach biologically relevant levels. This variability is particularly relevant when considering long-term supplementation or dietary enrichment with IX-containing products, such as hop extracts or certain types of beer.

Moreover, certain populations may be especially vulnerable to the estrogenic effects of 8-PN. Individuals with hormone-sensitive conditions—such as ER-positive breast cancer, endometriosis, or uterine fibroids—could theoretically experience adverse effects from even modest systemic exposure to phytoestrogens. Similarly, caution may be warranted for postmenopausal women undergoing hormone replacement therapy or for individuals taking medications that modulate ER activity. The long-term effects of chronic 8-PN exposure, whether through dietary IX or supplements, remain unknown and warrant further investigation.

On the other hand, the estrogenic activity of 8-PN may not be universally detrimental. In specific contexts—such as perimenopausal symptom management or osteoporosis prevention—it may provide therapeutic benefits. Indeed, some studies have explored the utility of 8-PN as a plant-based alternative to conventional hormone therapy, citing its high receptor affinity and SERM-like behavior. However, these applications must be weighed against the broader endocrine disruption potential, particularly when consumption is not medically supervised.

## 6. Conclusions

IX is a plant-derived compound of growing importance, whose biological effects reflect a complex balance between therapeutic potential and safety concerns. Preclinical studies indicate that it may beneficially influence glucose and lipid metabolism and exhibit anti-inflammatory effects, in part through interactions with the gut microbiota. However, its biotransformation to 8-PN, a compound with potent estrogenic activity, poses a significant limitation in assessing the safety of IX. An additional challenge is the interindividual variability of the gut microbiota, which influences the rate of this conversion and the level of exposure to the organism. Therefore, despite its promising properties, particularly in the context of metabolic disorders, further research is necessary on its long-term effects and safety in various populations, especially in hormone-dependent diseases. Further understanding of IX metabolism and the possibility of modulating its biological activity through structural modifications, such as complexation with metals, will also be crucial, as this could contribute to the development of safe and effective dietary and therapeutic applications.

## Figures and Tables

**Table 1:** The main enzymes, sites of action, and metabolites involved in the metabolism and biotransformation of isoxanthohumol (IX).

Stage	Enzyme/System	Location	Metabolite/Product	Ref.
Phase I (oxidation)	Cytochrome P450 (e.g., CYP1A2, CYP3A4)	Liver, small intestine	Hydroxylated IX derivatives	[17]
Phase II (conjugation)	UDP-glucuronosyltransferases (UGTs), sulfotransferases (SULTs)	Liver, intestine	IX-glucuronides, IX-sulfates	[18]
Epoxide/hydroxylation	Microbial monooxygenases, epoxidases	Colon microbiota	Hydroxy-IX, epoxy-IX	[19]

## Data Availability

No new data were created or analyzed in this study. Data sharing is not applicable to this article.

## Abbreviations

The following abbreviations are used in this manuscript:

IX	Isoxanthohumol
XN	Xanthohumol
UGT	UDP-glucuronosyltransferases
SULTs	sulfotransferases
8-PN	8-prenylnaringenin
DMX	O-desmethylxanthohumol
DDXN	O-desmethyl-α,β-dihydroxanthohumol
HMA	human-associated microbiota
MCF-7	human breast cancer cell line
A-2780	ovarian cancer cell line
HASMCs	human aortic smooth muscle cells
HSV1	herpes virus 1
BVDV	bovine viral diarrhea virus
SHIME	dynamic gut models
ER	estrogen receptor
ERα	estrogen receptor alpha
ERβ	estrogen receptor beta
PLGA	polylactic-co-glycolic acid
PEG	polyethylene glycol
RBA	relative binding affinity
BMD	bone mineral density
ZEN	zearalenone
LPS	lipopolysaccharide
DXN	α,β-dihydro-isoxanthohumol
TXN	tetrahydro-isoxanthohumol
hCG	human chorionic gonadotropin
StAR	steroidogenic acute regulatory protein
cAMP	cyclic adenosine monophosphate
SERM	selective estrogen receptor modulator
